# Non-target metabolomics revealed the differences between *Rh. tanguticum* plants growing under canopy and open habitats

**DOI:** 10.1186/s12870-021-02897-8

**Published:** 2021-02-27

**Authors:** Feng Xiong, Xiuqing Nie, Lucun Yang, Lingling Wang, Jingjing Li, Guoying Zhou

**Affiliations:** 1 CAS Key Laboratory of Tibetan Medicine Research, Northwest Institute of Plateau Biology, Xining, 810008 China; 2 College of Resources and Environment, University of Chinese Academy of Science, Beijing, 100049 China; 3 Key Laboratory of Tree Breeding and Cultivation of the State Forestry Administration, Research Institute of Forestry Chinese Academy of Forestry, Beijing, 100091 China; 4 Research Institute of Nature Protected Area Chinese Academy of Forestry, Beijing, 100091 China; 5 College of Life Sciences, Qinghai Normal University, Xining, 810008 China

**Keywords:** *Rheum tanguticum*, Habitats, Non-target metabolism, Morphology, Ecotypes

## Abstract

**Background:**

*Rheum tanguticum* (*Rh. tanguticum*) is an important traditional Chinese medicine plant, “Dahuang”, which contains productive metabolites and occupies wide habitats on the Qinghai-Tibet plateau. Plants occupying wide habitats usually vary in phenotypes such as in morphology and metabolism, thereby developing into different ecotypes. Under canopy and open habitats are a pair of dissimilar habitats which possess *Rh. tanguticum* plants. However, few studies have focused on the effect of habitats on *Rh. tanguticum* growth, particularly combining morphological and metabolic changes. This study focused on *Rh. tanguticum* plants growing in under canopy and open habitats where morphology and metabolism changes were quantified using non-target metabolism methods.

**Results:**

The obtained results indicated that the two dissimilar habitats led to *Rh. tanguticum* developing into two distinct ecotypes where the morphology and metabolism were simultaneously changed. Under canopy habitats bred morphologically smaller *Rh. tanguticum* plants which had a higher level of metabolites (22 out of 31) which included five flavonoids, four isoflavonoids, and three anthracenes. On the other hand, the open habitats produced morphologically larger *Rh. tanguticum* plants having a higher level of metabolites (9 out of 31) including four flavonoids. 6 of the 31 metabolites were predicted to have effect targets, include 4 represent for under canopy habitats and 2 for open habitats. Totally, 208 targets were connected, among which 42 were communal targets for both under canopy and open habitats represent compounds, and 100 and 66 were unique targets for under canopy superior compounds and open habitats superior compounds, respectively. In addition, aloe-emodin, emodin, chrysophanol, physcion, sennoside A and sennoside B were all more accumulated in under canopy habitats, and among which aloe-emodin, emodin, chrysophanol and physcion were significantly higher in under canopy habitats.

**Conclusions:**

This study determined that *Rh. tanguticum* growing in under canopy and in open habitats developed into two distinct ecotypes with morphological and metabolic differences. Results of network pharmacology study has indicated that “Dahuang” coming from different habitats, such as under canopy and open habitats, are different in effect targets and thus may have different medicinal use. According to target metabolomics, under canopy habitats may grow better “Dahuang”.

**Supplementary Information:**

The online version contains supplementary material available at 10.1186/s12870-021-02897-8.

## Background

*Rheum tanguticum* Maxim. ex Balf (*Rh. tanguticum*) belongs to the *Polygonaceae* family [1, 2]. A previous study reported that there are many secondary metabolites of *Rheum* species [3]. The dry roots of *Rh. tanguticum* have been listed as herbal medicine in the official pharmacopoeias of many countries due to its strong purgative power [4–6]. According to the *Chinese Pharmacopoeia* (2015), *Rh. tanguticum*, *Rh. palmatum* and *Rh. officinal* have been classified as “Dahuang”. “Dahuang” has been shown to have several effects throughout the long herbal utilization history with the main effects including purgative, clearing heat, detoxification, breaking blood stasis, and relieving jaundice [4]. More than 100 chemical components were reported from the three *Rheum* species [7]. In China, *Rh. tanguticum* is mainly distributed in Qinghai, Sichuan, and Gansu provinces and in the region to the east of the Tibet Autonomous Region at an altitude ranging from 2000 m to 4600 m. Studies have shown that the quality of *Rh. tanguticum* varies among different geological origins [8, 9], which can be attributed to environmental variations such as altitude [10] or other climate and soil factors [11].

Studies have reported that plants grown at specific habitats vary in their phenotypic performance [12, 13], with plants occupying a wider range of habitats having more variations in their phenotype [12, 14]. For example, a study reported variations in the leaf size of plants grown in a wide range of latitude due to temperature differences between the leaf and the air [15]. In addition to morphology, the chemotype also acts as a direct indicator of plant phenotype at the metabolomic level [16], and it reveals how the plants survive in a complex ecological environment [17]. Plant metabolomics provide a powerful tool for observing the enormous chemical diversity of plants and it helps in understanding the chemotypes [18]. An example is a study which reported metabolic differences in orange fleshed and white fleshed sweet potato ecotypes where 148 and 126 metabolites respectively were identified as the characteristic chemotype metabolites for the two ecotypes [19]. Similarly, five chemotypes of *Origanum vulgare* (L.) were identified using the metabolomics approach [20]. In addition, several studies have reported that different ecotypes of plants usually respond to environmental changes in different ways [21–23].

Growing plants under a canopy or in the open habitat has a significant effect on the morphological and metabolic phenotypes of the plants [24]. This can be attributed to the changes in environmental factors such as light conditions [25] and soil conditions [26]. Grapevines grown under canopy habitats were found to have a higher flower drop percentage and a lower level of some primary metabolites in its inflorescences [27]. A previous study reported that the content of LMP-HPM in non-adapted *Pteridium arachnoideum* was significantly higher in plants grown in a sun-exposed habitat than in those grown under a canopy habitat [28]. Generally, sunlight is an important factor that affects the growth and development of plants [29]. The photosynthetic process varies as a result of changes in light availability and light composition with a study reporting a lowered photosynthetic capacity of soybean plants grown in reduced light [30]. A previous study has shown that growing plants under canopy leads to light composition changes [29]. Light changes affect many of the physiological and morphological parameters in seedlings of tropical *Bauhinia lianas* [31], and the phenology, growth, and biomass of *Sacha Inchi* [32]. Another study suggested that these effects differ among species [33]. In addition, the phenotype differences in plants grown under canopy habitats and open habitats can also be contributed by soil condition differences [26]. This is because soil conditions such as soil nitrogen content [34, 35] and soil humidity [36] are also key factors that affect plant growth. Additionally, some properties such as wet and full organic compounds are beneficial to plants in adapting to growing under canopy shading [37].

Generally, *Rh. tanguticum* plants have a broad habitat where they are natively distributed in a series of habitats including under canopy environments and open lands such as grasslands [38, 39], with the habitat differences urging this species to exhibit different phenotypes. In this study, two ecotypes of *Rh. tanguticum* growing under-canopy-adapted and open-habitats-adapted were observed. Under-canopy-adapted plants were relative small and the roots were thinner. Currently, few studies have focused on the effects of habitats on the quality of *Rh. tanguticum*, and the metabolic changes between under canopy and open habitats *Rh. tanguticum* plants have not been elucidated.

Consequently, the aim of this study was to find out the differences between two ecotypes of *Rh. tanguticum* where the morphology and non-target metabolism of *Rh. tanguticum* from open and under canopy habitats were explored. Two specific habitats were introduced where *Rh. tanguticum* plants grown in both habitats were collected from Qinghai, Sichuan, and Gansu provinces. The hypothesis for the study was that the two distinct habitats bred two ecotypes of *Rh. tanguticum* with regards to the morphology and the chemotype. The results obtained from the two specific habitats can lead to researchers asking more detailed questions such as the influence of sunlight or soil conditions, thereby leading to an understanding of the potential drivers of the secondary metabolomics, and its connection with the observed morphological changes.

## Results

### Morphological differences of *Rh. tanguticum* plants growing in two different habitats

**Fig. 1:**
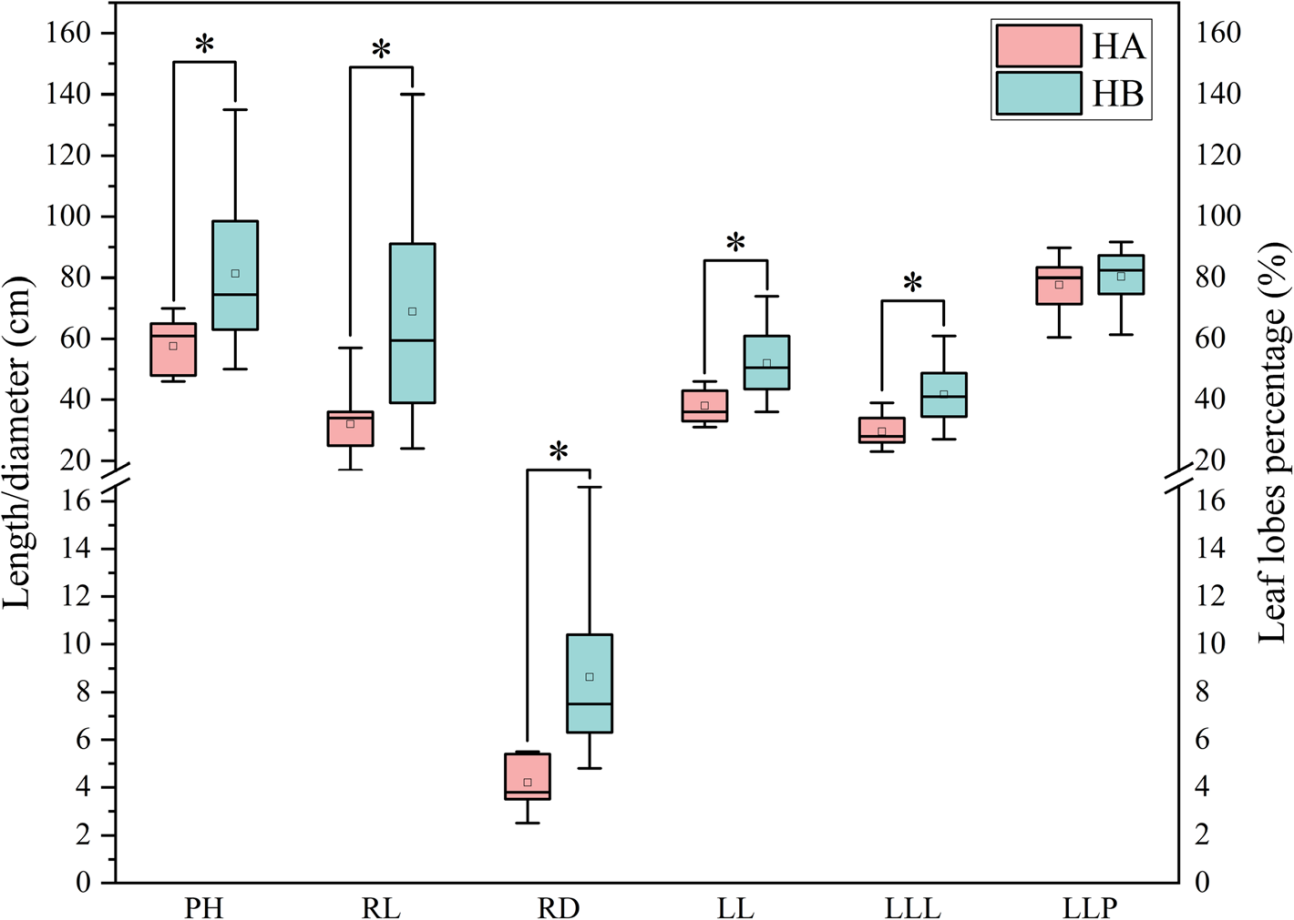
Differences in plant traits of *Rh. Tanguticum* between HA and HB. Red box represents the HA and the blue box represent HB. The top and bottom of each box represent 25th and 75th percentiles, the center line indicates the median, and the little hollow squares indicates mean value. The extents of the whiskers show the extent of the data while the asterisk represents significant difference between two groups. Plant traits include plant height (PH), root length (RL), root diameter (RD), leaf length (LL), leaf lobes length (LLL), and the leaf lobes percentage (LLP)

Significant differences were found among plant height, root length, root diameter, leaf length and leaf lobes length, but not found in leaf lobes percentage (Fig. 1). It was evident that the plant and root sizes were significantly lower in HA (under canopy habitat) than in HB (open habitat) indicating that the habitat has an effect on the growth of *Rh. Tanguticum*. However, there was no significant difference on the two habitats when the leaf lobes percentage was analyzed. It is worth noting that the leaf lobes percentage is usually used to indicate different species in the *Rheum* family.

### Overall metabolic profiling of the *Rh. tanguticum* samples using UPLC-MS-MS

Thirty root samples of *Rh. tanguticum* plants collected from two different habitats distributed in Qinghai, Gansu, and Sichuan provinces (Table 1) were analyzed. Three sites were selected for each habitat, and five plants were excavated from each site. The plants were then used for non-target metabolomics analysis to investigate the metabolomic differences in *Rh. tanguticum* plants obtained from the two different habitats. Plants were collected from one site in August 2016 while the other two sites were collected in August 2018 in both under canopy and open habitats to minimize the influence of storage time. In addition, there was no significant difference on the average relative altitude of the selected sites in both habitats where the relative altitude was calculated as altitude + (latitude - 30)*111 [40]. The metabolites were extensively extracted and analyzed using UPLC-QTOF-MS^E^ in both positive and negative ion mode.

**Table 1:** Information of sample sites

Site number	Latitude	Longitude	Altitude	Relative altitude	Location	Habitat	Sampling date
HA-1	34°30.772′	103°30.032′	3173	3673.9282	Gansu province	Under canopy	2018.8.23
HA-2	33°34.072′	103°10.735′	3321	3717.0332	Sichuan province	Under canopy	2018.8.28
HA-3	34°47.082′	100°48.859′	3381	3912.1017	Qinghai province	Under canopy	2016.8.14
HB-1	33°59.714′	101°41.145′	3634	4077.4709	Gansu province	Open	2018.8.24
HB-2	33°27.753′	102°29.361′	3465	3849.3431	Sichuan province	Open	2018.8.27
HB-3	35°04.411′	101°49.037′	3545	4108.1604	Qinghai province	Open	2016.8.15

HA: under canopy habitat, and HB: open habitat

The total ion chromatograms of both positive and negative scan modes are shown in Fig. S1. A total of 22,310 and 15,290 ion peaks were detected in positive and negative modes respectively. The peaks were reduced to 19,828 and 12,218 peaks respectively when the missing values were compared with QC sample followed by the filling up of the missing values of raw data using half of the minimum value. The metabolites were identified by scoring according to accurate mass, MS/MS fragments, and isotope distribution where each item accounted for 20 scores. The peaks that scored more than 45 (total of 60) were reserved. Finally, 410 and 302 peaks were annotated in positive and negative modes, respectively.

### PCA of the two different habitats

**Fig. 2:**
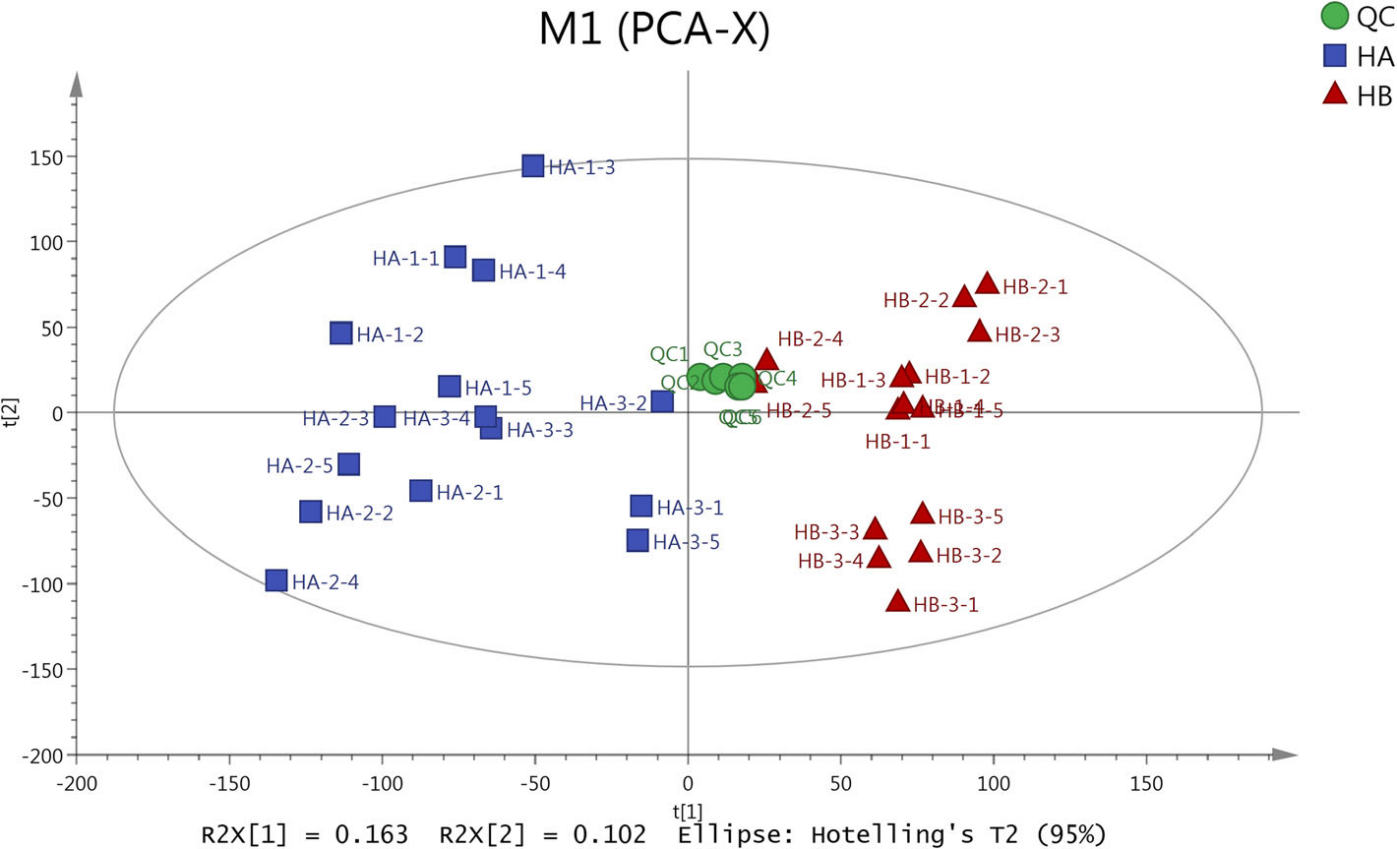
PCA for all samples

Multivariate data analysis was conducted to analyze the metabolomics profile of *Rh. tanguticum* plants collected from the two different habitats. Principle component analysis (PCA) was first used to reveal the overview classification of the samples where 7-fold cross-validation was used to test the model. The first principle accounted for 16.3% variety while the second principle accounted for 10.2% of total variety. The QC samples clustered into one small set near the coordinate origin indicating that the accuracy of the experiment could be guaranteed. The samples of HA and HB were separated into different groups (Fig. 2) indicating the significant differences in the metabolites of *Rh. Tanguticum* plants obtained from the two different habitats.

### HCA of the two different habitats

**Fig. 3:**
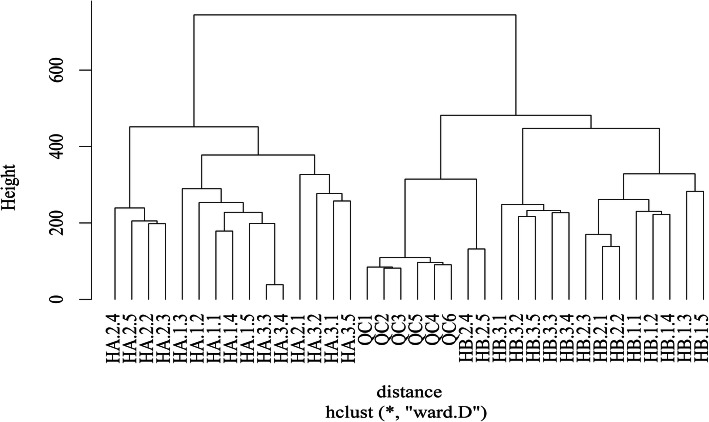
HCA results of the samples

Hierarchical clustering analysis (HCA) was performed to further determine the cluster pattern of the metabolites. The obtained HCA results indicated that the HA samples were clustered as one group while the HB samples together with QC samples were clustered as the other group (Fig. 3). QC samples in the second set were clustered as a small set which was similar with PCA results.

### OPLS-DA of the two different habitats

**Fig. 4:**
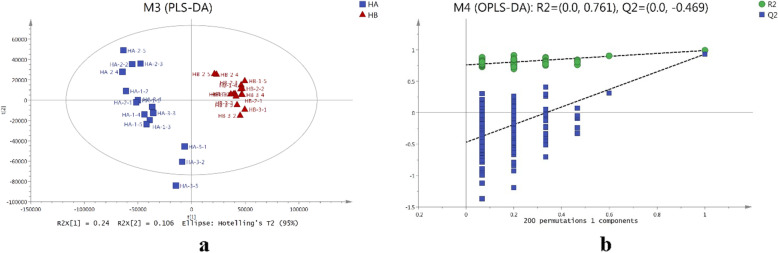
OPLS-DA results of the samples

Supervised OPLS-DA approach was used to maximize sample separation. The obtained OPLS-DA results indicated that HA samples were clustered on the left part of the X-axis while HB samples were clustered on the right part of the X-axis (Fig. 4a). 7-fold cross validation was applied where R^2^ and Q^2^ were 0.761 and − 0.469 respectively (Fig. 4b), which indicated that there was no over-fitting in the model. This was followed by conducting response permutation testing 200 times. The obtained R^2^Y of totally three compounds and Q^2^ were 0.991 and 0.934 respectively, which indicated that the model was able to explain the differences of the samples.

VIP values were extracted from the OPLS-DA model while *p* values were calculated using ANOVA and Student’s t-test to further identify the key metabolites in the two habitats. 31 metabolites were filtrated out as the different metabolites when VIP > 4 and *p* value < 0.05 were used, among which 13 were detected at positive ion mode while 18 were detected at negative ion mode.

### Heatmap of the metabolites from HA and HB

**Fig. 5:**
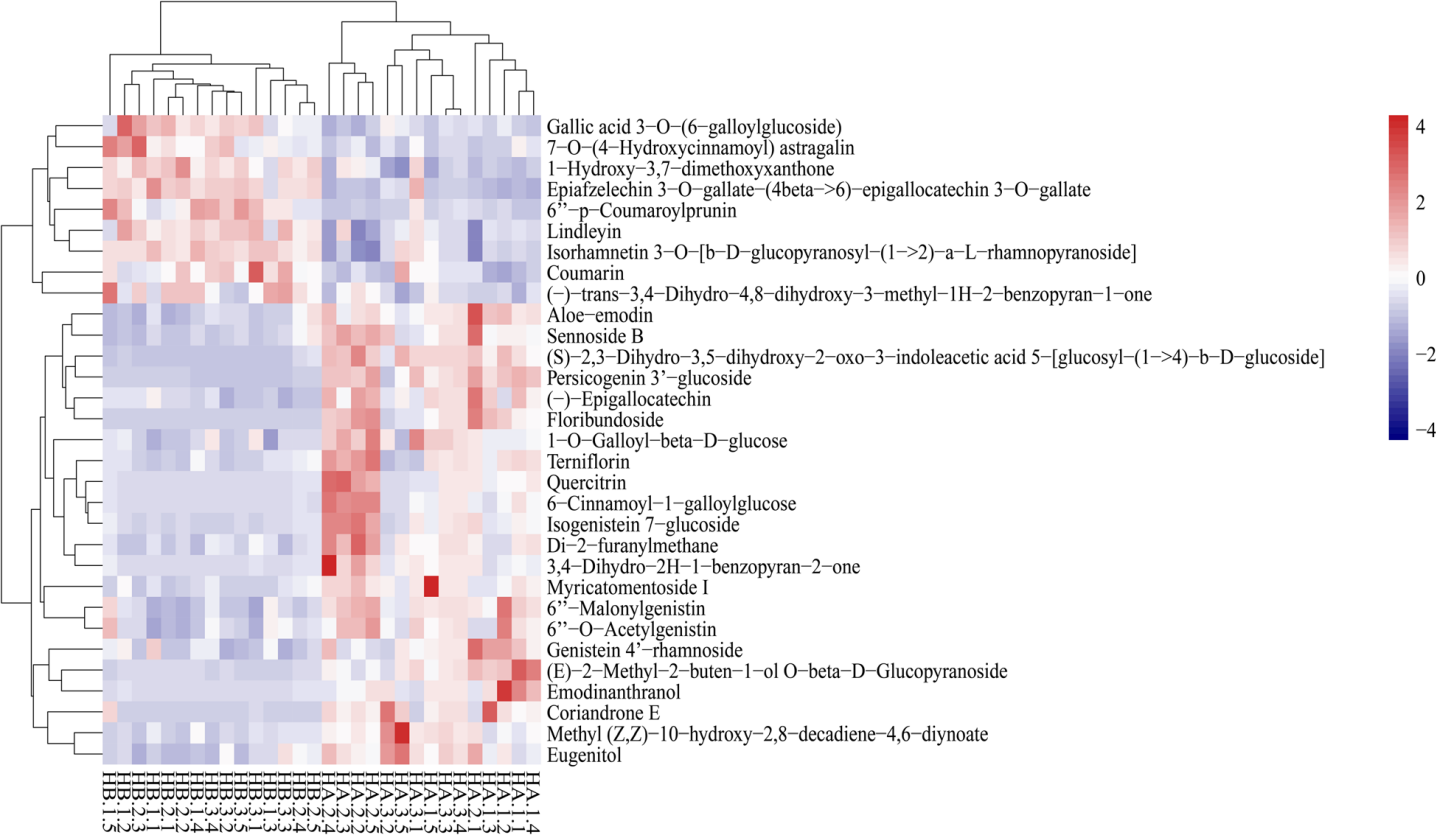
The hierarchical cluster analysis of differential metabolomics in samples of the two different habitats. The red color represents higher metabolite contents while the blue color represents lower contents

A heatmap of the 30 samples verses 31 metabolites (VIP > 4 and *p* value < 0.05) was plotted to create a better overview of the differences of metabolites between the two different habitats (Fig. 5). According to the color series of all samples, samples within the same habitat share similar contents of the metabolites where nine metabolites were higher at HB while 22 metabolites were higher at HA (Fig. 5). The nine metabolites higher at HB include Gallic acid 3-O-(6-galloyglucoside), 7-O-(4-Hydroxycinnamoyl) astragalin, 1-Hydroxy-3,7-dimethoxyxanthone, Epiafzelechin 3-O-gallate-(4beta- > 6)-epigallocatechin 3-O-gallate, 6″-p-Coumaroylprunin, Lindleyin, Isorhamnetin 3-O-[b-D-glucopyranosyl-(1- > 2)-a-L-rhamnopyranoside], Coumarin, and (−)-trans-3,4-Dihydro-4,8-dihydroxy-3-methyl-1H-2-benzopyran-1-one. On the other hand, the 22 metabolites higher at HA include Aloe-emodin, Sennoside B, (S)-2,3-Dihydro-3,5-dihydroxy-2-oxo-3-indoleacetic acid 5-[glucosyl-(1- > 4)-b-D-glucoside], Persicogenin 3′-glucoside, (−)-Epigallocatechin, Floribundoside, 1-O-Galloyl-beta-D-glucose, Terniflorin, Quercitrin, 6-Cinnamoyl-1-galloylglucose, Isogenistein 7-glucoside, Di-2-furanylmethane, 3,4-Dihydro-2H-1-benzopyran-2-one, Myricatomentoside I, 6″-Malonylgenistin, 6″-O-Acetylgenistin, Genistein 4′-rhamnoside, (E)-2-Methyl-2-buten-1-ol O-beta-D-Glucopyranoside, Emodinanthranol, Coriandrone E, Methyl (Z,Z)-10-hydroxy-2,8-decadiene-4,6-diynoate, and Eugenitol.

## Discussion

### Morphology differences of *Rh. tanguticum* from the two habitats

Six morphological characteristics of *Rh. tanguticum* were explored in this study with results showing that five of them had significant differences between the two habitats (Fig. 1). These differences indicated that there were two ecotypes morphologically expressed between the two dissimilar habitats. Plant growth was higher in open habitats with the plants having significantly larger leaves and more roots than under canopy habitats. UV-B radiation was significantly higher when the high altitude on the Qinghai-Tibet plateau was considered. Therefore, the *Rh. tanguticum* plants might have developed protective mechanisms such as reinforced photosynthesis and water use efficiency to protect them from UV-B and light damages [41]. However, the sunlight was filtered by canopy plants in the under canopy habitats and thus the UV-B light, blue light, red light and red to far red light ratio were all decreased [29]. All these changes in light quality and quantity can scale down photosynthesis. Therefore, *Rh. tanguticum* plants growing in the under canopy habitats were significantly smaller than those in open habitats. These results are consistent with results obtained from a study done on soybean where the general photosynthetic capacity was decreased despite the reduced light leading to higher light capture ability of soybean [30].

In addition, a study has reported that the soil properties are affected in native under canopy habitats which could affect the growth and development of plants [26]. The obtained results indicated that soil humidity, soil total nitrogen content, and soil organic carbon content were higher at under canopy habitats (Fig. S3), and the differences in soil organic carbon and soil total nitrogen contents between the two habitats were significant. Under canopy habitats provide more nutritious and moist soil than open habitats, which contributes to the shade tolerance of plants [37] and, therefore, widens the niche of *Rh. tanguticum*.

### Chemotypes of *Rh. tanguticum* from two habitats

The samples could be separated into two groups based on PCA (Fig. 2) and HCA (Fig. 3) results, which indicated the metabolism differences between the two ecotypes. The results support our hypothesis that the two habitats breed two different chemotypes in addition to different morphology. Habitat induced chemotypes have been widely reported in plants. For example, American ginseng grown in wild and field habitats were significantly different in their metabolisms [42]. The medicinal compounds of *Rh. tanguticum* have been widely studied because it is used as a medicinal herb. Previous studies have indicated that the medical compounds are associated with altitude [10], season [43, 44], or some environmental factors [11]. However, there was little compatibility among the various studies indicating that a single factor may not be attributed to the variation in quality of *Rh. tanguticum*, and that the environmental factors causing the most habitat differences should be put forward for further studies on how rhubarbs achieved better quality. *Rh. palmatum* complex, including *Rh. tanguticum, Rh. palmatum, and Rh. officinal* together with few other species, have been found to relate with geological origins with regards to the metabolites [8, 45], which revealed different chemotypes of different geological origin in Qinghai province, Gansu province, and Sichuan province. However, although the administration region may uncover climate gradient in a relative large scale, it cannot reveal the exact habitat affection such as under canopy or open. In addition, there was no connection between the chemotype and the morphological variation. In this study, the morphology and metabolites revealed a simultaneous pattern in response to habitat change. Our previous study also revealed that the total anthraqunones content differed at under canopy and grassland habitats [38].

In this study, canopy shade was attributed to the light condition changes in the plants growing in under canopy habitats [29]. The soil conditions such as soil humidity and nutrients also contributed to the changes. Canopy shade can lead to low UV-B radiation, low blue and red light, and a decreased red light to far red light ratio. The red and blue light is absorbed by photosynthetic pigments of plants growing under canopy [29]. The under canopy herbal plants have evolved shade tolerance strategies such as decreasing self-shading [46], increasing leaf expansion [47], specific leaf area, and photosystem II:I ratios [48, 49]. This is because they cannot avoid the shade habitats since they are unable to outcompete the woody plants. Soil humidity, soil total nitrogen content, and soil organic carbon content were also different where they were significantly higher at under canopy habitats with the exception of soil humidity (Fig. S3). These results may also lead to metabolomic differences between the two ecotypes [17].

### Significantly changed metabolites

31 metabolites were filtered out as the significantly changed metabolites using VIP and *p* values. The results indicated that 22 of the 31 metabolites were more accumulated in under canopy habitats while only nine metabolite were more accumulated in open habitats. The significantly higher number of secondary metabolites indicated that the plants were growing in a stressful environment [50]. Both the under canopy and open habitats ecotypes of *Rh. tanguticum* were facing distinct environmental stresses. Specifically, most of the light was filtered by upper canopy in under canopy habitats which lead to lower photosynthesis rates when compared with open habitats [29], thus it might be the most stressful factor. On the other hand, soil nutrients or soil humidity were limited for open habitats (Fig. S3), and thus they might be the most stressful factors.

In total, 22 metabolites were more accumulated at under canopy habitats with most of them belonging to flavonoids, isoflavonoids, and anthracenes. In addition, nine metabolites including four flavonoids were higher at open habitats. The results indicated that flavonoids were the most changed class of metabolites. Several studies have focused on the flavonoids in leaves because they contain UV absorbing compounds which protect the leaves against the deleterious effects of UV radiation [51, 52]. However, different types of flavonoids may have distinct responses to light change, which may be attributed to their different functions. Full light induced chlorotic leaves in a study done on tea plants where the flavonoids with ortho-dihydroxylated B-rings increased while total flavonoids decreased [53]. Generally, leaves have usually been used as the study object due to the UV protective mechanism of flavonoids. Results obtained in this study indicate that flavonoids in the roots also vary between under canopy and open habitats suggesting that changes in light conditions also influences the flavonoids in roots. And in this study, flavonoids with relatively simple structure accumulated more in under canopy habitats, such as terniflorin, quercitrin, floribundoside, while complex flavonoids in structure accumulated more in open habitats, such as Isorhamnetin 3-O-[b-D-glucopyranosyl-(1- > 2)-a-L-rhamnopyranoside], 6″-p-Coumaroylprunin, Epiafzelechin 3-O-gallate-(4beta- > 6)-epigallocatechin 3-O-gallate. The under canopy habitats were characterized by lower light quality and quantities, and wet soil enriched with organic compounds. Therefore, the flavonoids are also thought to be associated with other environmental stresses such as water, salt, nitrogen deficiency, and cold [54].

Among the 31 most changed metabolites, aloe-emodin was listed in the Chinese Pharmacopoeia [4] as one of the main medical component used for judging the quality of “Dahuang”, and sennoside B was also an common used index to accesses quality. Both components were more accumulated in under canopy habitats, which suggests that under canopy habitats may be beneficial for promoting the medicinal quality of *Rh. tanguticum*. The results are consistent with a previous study which reported that aloe-emodin was higher at under canopy or forest edge habitats [38]. In addition, the results were consistent with another study which reported that sennoside B in senna (sona cultivar) was higher in medium light conditions [55]. However, the other cultivar of senna (ALFT cultivar) had the highest sennoside B content at full sunlight condition which suggests that different species or even cultivars have distinct responses to environmental changes in the metabolomics level. Besides, quercitrin had been uncovered to have bioactive such as antioxidant, anti-inflammation and enhance osteoblast [56]. It accumulated more in under canopy habitats, and had also been identified and quantified in seeds of *Rh. palmatum* [57]. However, lindleyin [58] and coumarin [59], which were accumulated higher at open habitats, had been identified from rhubarb and were demonstrated to beneficial for protect neuro and antioxidant or anti-inflammatory and have a few pharmacological effects [58, 60, 61]. The difference in metabolites between under canopy and open habitats may reveal that distinct habitats grows “Dahuang” differed in medical efficacy.

### Target metabolic analysis of *Rh. tanguticum*

**Fig. 6:**
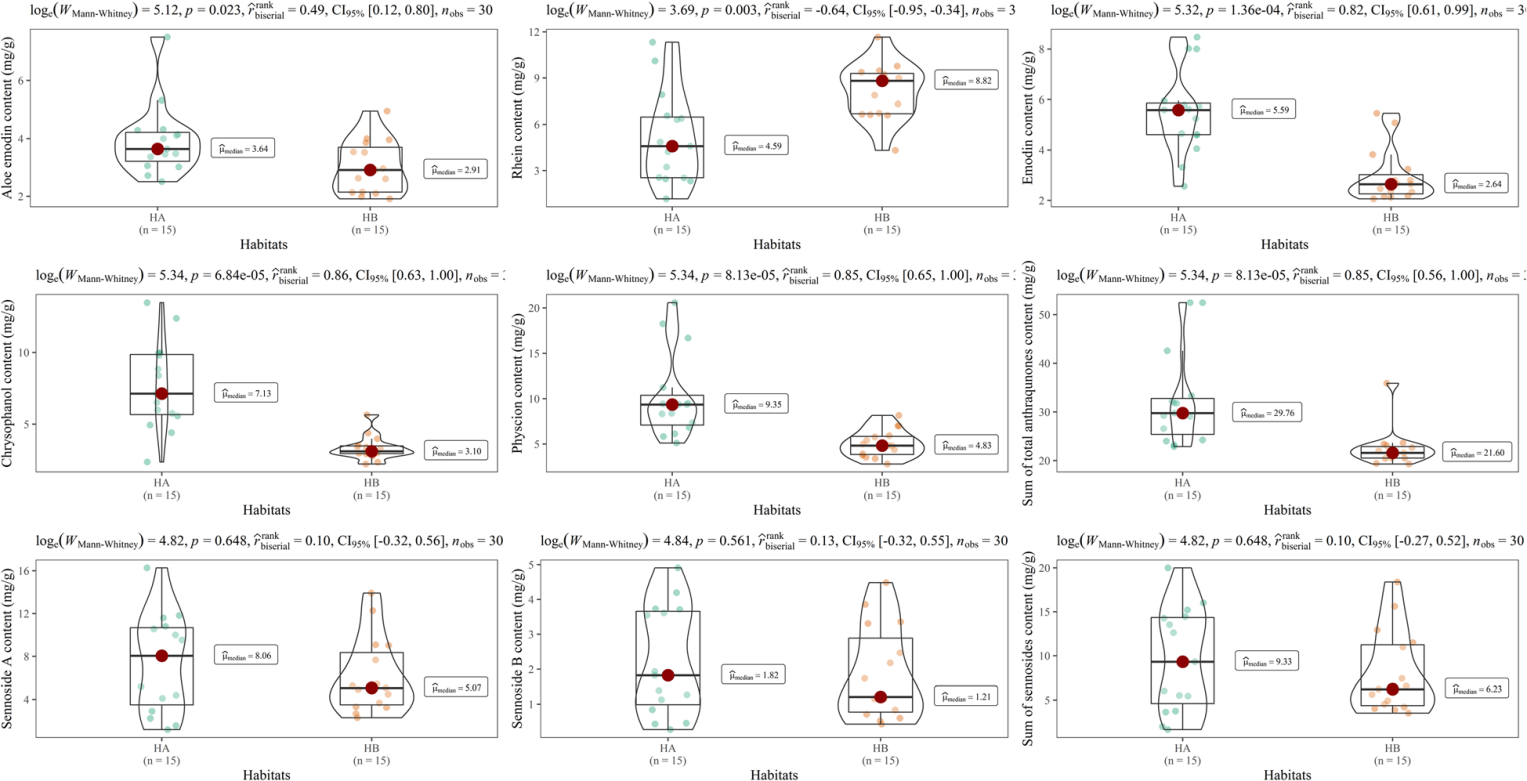
Targeted metabolites comparison between under canopy (HA) and open (HB) habitats

As an important medical herb, some components had been uncovered to have varies pharmacological actions, and some compounds, such as aloe-emodin, rhein, emodin, chrysophanol, physcion, sennoside A and sennoside B were commonly used to access quality of “Dahuang” [4, 5]. To evaluate differences in medicinal value, targeted metabolic analysis was employed to quantify contents of the 7 compounds above (Fig. 6). The results revealed that, except for rhein, the 6 effective compounds were all accumulated more in under canopy habitat plants (Fig. 6). Aloe-emodin, emodin, chrysophanol and physcion contents were significantly higher in under canopy habitats plants, and also, the sum of total anthraqunones was accumulated more in under canopy habitats plants, significantly. Sennoside A, sennoside B and the sum of sennosides were higher in under canopy habitats, while the differences were not significantly. Rhein was significantly higher in open habitats. These results were in consistent with non-target metabolites results (Fig. 5). The results indicated that under canopy habitats was beneficial for *Rh. tanguticum* to accumulate more effective compounds. In traditional Chinese medicine, “Dahuang” from Sichuan was good in quality, which may due to there are lots of mountains and most of “Dahuang” were grown in under canopy habitats.

### Network pharmacology analysis of the differences metabolites

**Fig. 7:**
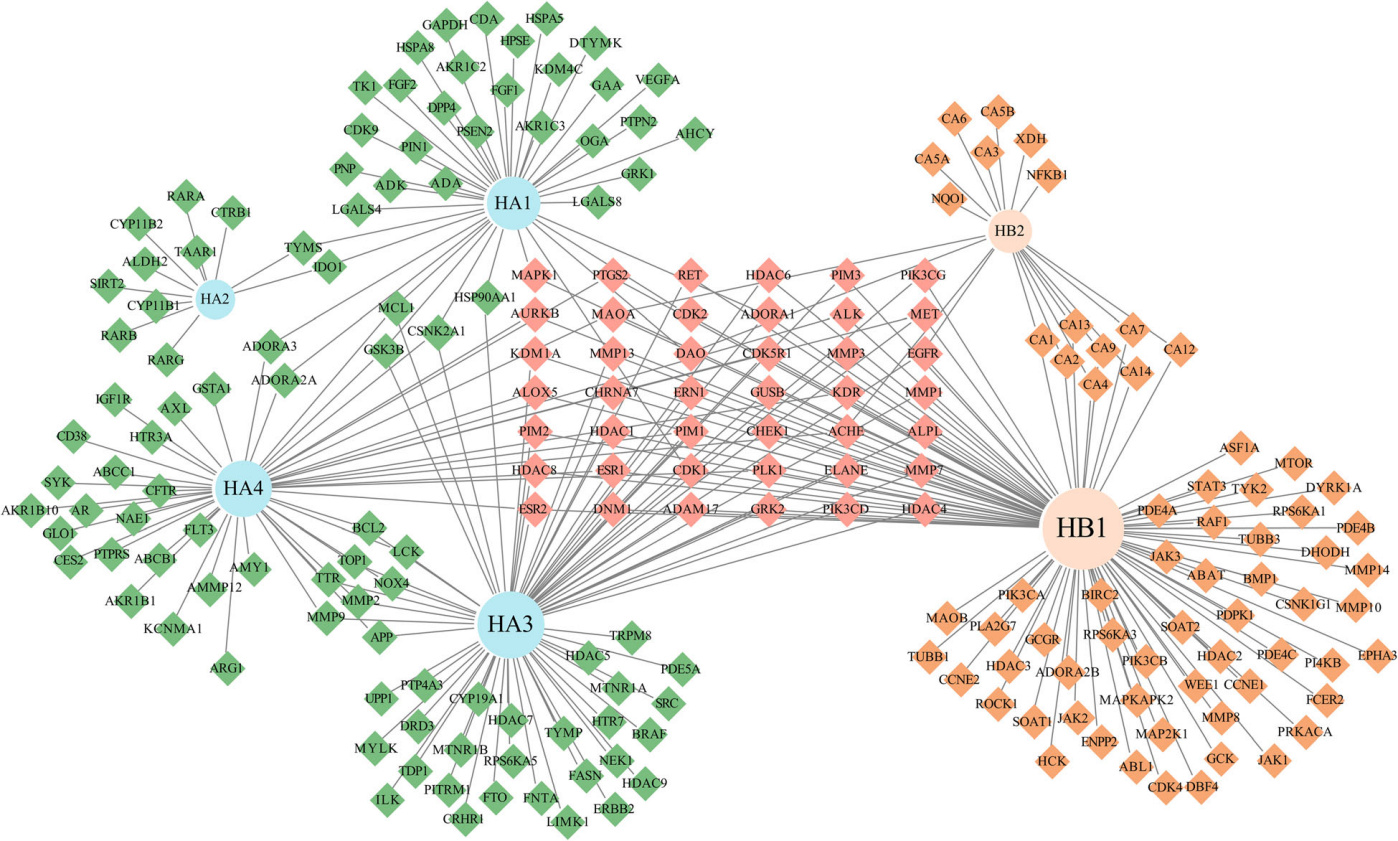
Network pharmacology analysis of difference metabolites. Diamons were predicted targets and circles were compounds. The light pink diamons were the common targets, and the green and orange diamons were represented under canopy habitats higher compounds and open habitats higher compounds targets, respectively. Light blue and light orange circle were represented under canopy compounds and open habitats compounds, respectively. Degree were performed by the size of symbols

Traditional Chinese medicines often performed a complex therapeutic efficacy [62]. To investigate therapeutic efficacy differences between “Dahuang” from different environments. A further network pharmacology study was conducted on the 31 significantly different metabolites, which could reveal the difference in effect targets [63]. After the ADME (absorption, distribution, metabolism, and excretion) filter process, 12 metabolites were chosen for target prediction, and 6 of them were predicted to have potential targets with probability larger than 0. Among them, 4 metabolites were accumulated higher in under canopy habitat plants ((E)-2-Methyl-2-buten-1-ol, 3,4-Dihydro-2H-1-benzopyran-2-one, Emodinanthranol, and Eugenitol), and 2 metabolites were accumulated higher in open habitat plants (1-Hydroxy-3,7-dimethoxyxanthone and Coumarin) (Fig. 7; Table S1). Except for 42 common targets predicted by both two habitats compounds, the 4 compounds accumulated more in under canopy habitats pointed to 100 unique targets, and the 2 compounds accumulated more in open habitats have 66 unique targets. HB1 (1-Hydroxy-3,7-dimethoxyxanthone) was the metabolites with the largest degree, and followed by HA3 (Emodinanthranol). This result pointed out that growth habitat performed a critical role in shaping therapeutic efficacy of traditional Chinese medicines [64, 65].

## Conclusion

In this study, a non-target metabolomics approach combined with morphological observation were applied to examine morphological and metabolic differences of *Rh. tanguticum* growing at a pair of dissimilar habitats, under canopy and open habitats. It was revealed that morphology and metabolite were simultaneously changed between two habitats, which decided *Rh. tanguticum* plants into two ecotypes. Five of the six indicators of morphology revealed significant change between plants growing in different habitats, and under canopy habitats bred plants smaller in morphology compared to open habitats. Totally, 410 and 302 peaks were annotated respectively in positive and negative mode, and 31 of them were determined as significantly changes metabolites. *Rh. tanguticum* plants growing in under canopy habitats had higher levels of 22 of the 31 metabolites, while plants growing in open habitats had higher levels of the 9 of the 31 metabolites. 12 of the 31 metabolites were pass through the ADME filter, and finally 6 of them were predicted to have effect targets with probability larger than 0. Network study revealed that Emodinanthranol and 1-Hydroxy-3,7-dimethoxyxanthone are of higher degree in response of under canopy and open habitats, respectively. Aloe-emodin, emodin, chrysophanol, physcion, sennoside A, and sennoside B were accumulated more in under canopy grown plants, which indicated that under canopy may bred better *Rh. tanguticum* in quality as medicinal herb. This study may provide valuable information in both morphological and metabolomics to understand habitats effects on the important traditional herbal medicine *Rh. tanguticum*.

## Methods

### Plant materials and sample preparation for analysis

**Fig. 8:**
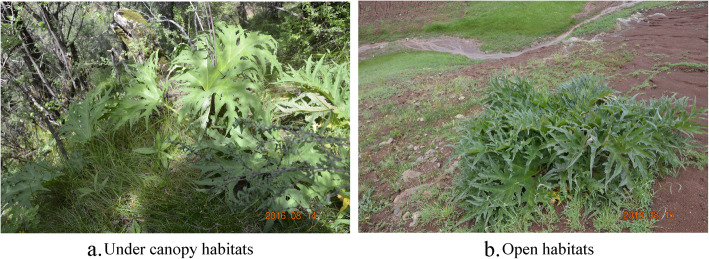
*Rh. tanguticum* in the (**a**) under canopy habitats and (**b**) open habitats

Roots of *Rh. tanguticum* plants were collected from Qinghai, Gansu, and Sichuan provinces. Under canopy and open habitats were selected as the sampling sites in each province where five whole *Rh. tanguticum* plants were excavated from each site for further analysis (Fig. 8). Samples were collected from August to September in a period ranging from 2016 to 2018 with the date of collection and geographical information being shown in Table 1. Plant height, root length, root diameter, leaf length, length of leaf lobes, and percentage leaf lobes (leaf lobes length to leaf length ratio) were measured after excavation of the plants*.* All samples were identified by Professor Guoying Zhou, and the roots were washed and air dried. The specimens were then stored at the herbarium in Northwest Institute of Plateau Biology, Chinese Academy of Science.

### Chemicals and reagents

Chemicals and solvents used in the study are all of HPLC grade. Water, acetonitrile, methanol, and formic acid were purchased from CNW Technologies GmbH (Düsseldorf, Germany), while L-2-chlorophenylalanine was purchased from Shanghai Hengchuang Bio-technology Co., Ltd. (Shanghai, China). Reference standards of emodin, aloe-emodin, rhein, chrysophanol, physcion, sennoside A, and sennoside B were used for contents calculate based on the methods described by our previous study [66].

### Soil properties

The soil humidity was measured using a portable TDR-100 soil moisture probe (Spectrum Technologies, Inc., Plainfield, IL, USA). Soil samples were collected alongside the plant roots at a depth of 0–10 cm. Air-dried soil samples were sieved through a 2-mm mesh and then ground to fine powder. The total nitrogen content of the dry soil samples was determined using the semimicro-Kjeldahl method [67] while the soil organic carbon content was determined using the Walkley-Black method [68].

### Untargeted UPLC-MS extraction and analysis

80 mg of the plant sample powder was weighed and introduced into a 1 mL solvent containing methanol and water (7:3 V/V ratio). This was followed by the addition of 20 μL of L-2-chlorophenylalanine (0.3 mg/mL) in methanol, and 20 μL of C-17 (0.01 mg/mL) in methanol which acted as the internal standard. Two small steel balls were then added followed by pre-cooling at − 20 °C for 2 min before they were put into an automatic sample rapid grinder (JXFSTPRP-24/32, Shanghai, China) at 60 Hz for 2 min. The samples were then placed in an ultrasonic cleaner (SB-5200DT, Ningbo, Zhejiang, China) for 30 min followed by cooling at − 20 °C for 20 min. The samples were then centrifuged at 13000 rpm (TGL-16MS, Shanghai, China) for 10 min at 4 °C. A syringe was used to suck out 200 μL of the supernatant, and the supernatant was then filtered through a 0.22 μm organic membrane into an LC sample injection vial and stored at − 80 °C. All the reagents used for the extraction were pre-cooled at − 20 °C before use. On the other hand, all samples were mixed to a quality control (QC) sample using the same volume, and all the QC samples had the same volume as the samples used.

UPLC-QTOF-MS^E^ analysis was performed using an ACQUITY UPLC system (Waters Corporation, Milford, MA, USA) and a Waters Xevo G2-XS QTof mass spectrometer System (Waters Corporation, Milford, MA, USA) coupled with an electrospray ionization (ESI) interface where the results were detected in both ESI positive and ESI negative ion modes. The UPLC system was equipped with an ACQUITY UPLC BEH C_18_ column (1.7 μm, 2.1 × 100 mm). The mobile phase composed of mobile A (0.1% formic acid in water, v/v) and mobile B (0.1% formic acid in acetonitrile, v/v). The elution condition was set as: 0 min, 1% B; 1 min, 5% B; 2 min, 25% B; 30 min, 60% B; 3.5 min, 60% B; 7.5 min, 90% B; 9.5 min, 100% B; 12.5 min, 100% B; 12.7 min, 1% B, and 16 min, 1% B. The flow rate was set at 0.4 mL/min while the column temperature was 45 °C. The injection volume was 2 μL and all samples were kept at 4 °C throughout the analysis.

The spectrum conditions were: source temperature, 120 °C; source offset, 80 V; reference capillary voltage, 2.5 kV; cone voltage, 40 V; desolvation gas temperature, 450 °C; desolvation gas flow, 800 L/h; and cone gas flow, 50 L/h. Additionally, the capillary voltage for the negative mode was -2 kV while the capillary voltage for the positive mode was + 3 kV. The collision dissociation gas used was argon (99.999%) while the desolvation and cone gas was nitrogen (> 99.5%). The data was acquired from 50 m/z to 1000 m/z in the MS^E^ centroid full scan mode by rapidly switching from a low energy (CE 6 eV) scan to a high energy (CE ramp 20-35 eV) scan with the scan rate being 0.1 s/scan. The external reference for lock mass correction was infused at a flow of 5 μL/min through the reference probe for 30s each. Leucine- enkephalin, performed as acetonitrile/water/formic acid (50:49.9:0.1, v/ v/v) in 250 ng/mL standard solution, was used as the external reference. The QC samples were injected for every 10 samples to enhance the data repeatability.

### Untargeted UPLC data processing

The raw data obtained from UPLC-MS was collected using the UNIFI 1.8.1 (Waters Corporation, Milford, MA, USA) and then imported to the Progenesis QI software (Nonlinear Dynamics, Newcastle, UK). The parameters were set as: 5 ppm for precursor tolerance, 10 ppm for fragment tolerance, and 0.02 min as the retention time (RT) tolerance. In addition, the internal standard detection parameters were employed for perk RT alignment while the noise level and minimum intensity were set at 10.00 and 15% of base peak intensity respectively to eliminate noise peaks. As a result, a 3D matrix expressed with m/z, peak RT, and peak intensities was obtained. Additionally, peaks with missing values larger than 50% were excluded. The internal standard was used for data QC (reproducibility). Metabolites were identified using the Progenesis QI automatic based on the accurate mass, MS/MS fragments, and isotope label by searching HMDB (http://www.hmdb.ca/), Lipidmaps (http://www.lipidmaps.org/), and METLIN (http://metlin.scripps.edu) public databases.

### Targeted metabolites analysis

Targeted metabolites analysis was conducted by an Agilent 1260 system which has been described in our former study [66]. Columns of Unitary C18 (4.6 × 250 mm, 5 μm, Acchrom) and Eclipse Plus C18 (250 mm × 4.6 mm, 5 μm, Agilent) were used. Mixed standard solutions of five anthraqunones, including emodin, aloe-emodin, rhein, chrysophanol and physcion (31.2 μgmL^− 1^_,_ 38.8 μgmL^− 1^_,_ 22 μgmL^− 1^_,_ 23.4 μgmL^− 1^_,_ and 23.4 μgmL^− 1^), and mixed standards of sennoside A and sennoside B (150 μgmL^− 1^ and 84mgmL^− 1^) were made. Peak area was used to calculate contents by comparing to standard solutions.

### Data analysis

The data containing both negative and positive ions was analysed using the SIMCA software package (version 14.0, Umetrics, Umeå, Sweden). Principle component analysis (PCA) was used to reveal the overview classification of the samples while orthogonal projections to latent structures discriminant analysis (OPLS-DA) was used to determine the maximum separation of HA and HB samples. 7-fold cross validation and response permutation testing (RPT) were applied to avoid over-fitting. R^2^ and Q^2^ were calculated as 0.761 and − 0.469 after 200 times of RPT indicating that the model was accurate. In addition, univariate analysis was conducted to further confirm the significantly different metabolites. The *p* value and the fold change value were obtained using student’s t-test in combination with fold change analysis and the results were visualized using a volcano figure (Fig. S2). Meanwhile, the variable importance for the projection (VIP) value together with the *p* value were used to select the different components. The metabolites with a VIP value of > 4.0 and *p* < 0.05 were considered relevant for group discrimination [69].

One-way analysis of variance (ANOVA) was used with Turkey test to compare mean values for plant morphological analysis with the significant level being set as 0.05. One-way ANOVA was also used to analyze the soil properties. Targeted metabolites were compared between under canopy and open habitats according to Mann-Whitney nonparametric test.

Network study was conducted by searching the 31 most changed metabolites. Swiss ADME (http://www.swissadme.ch/) [70] was used to filter the metabolites based on their ADME (absorption, distribution, metabolism, and excretion) assessments. Metabolites with high gatrointestinal absorption index (GI absorption) and at least three ‘yes’ in five druglikeness indexes (they were Lipinski, Ghose, Veber, Egan and Muegge, respectively) were left for the target prediction. Then, Swiss Target Prediction tool was employed to predict potential gene targets of the filtered metabolites from Swiss ADME tool [71]. Targets with probability larger than 0 were used in the network contribution with their common name. At last, network files contained 6 metabolites and 208 targets were imported into the Cytoscape 3.8.2 software for network analysis. The network figure was contributed by Cytoscape 3.8.2 [72].

## Supplementary Information

**Additional file 1: Fig. S1**: Total iron current chromatograms of metabolomics analysis in positive scan mode (a) and negative scan mode (b). **Fig. S2**: Volcano plots (log10 fold change vs. –log10 P -value). **Fig. S3:** Soil humidity, soil organic carbon contents and soil total nitrogen contents in the under canopy and open habitats. The top and bottom of each box represent 25th and 75th percentiles, the center line indicates the median, and the little hollow squares indicates mean value. The extents of the whiskers show the extent of the data. The asterisk represent significant difference between two groups. **Table S1** Compounds accumulated in different habitats

**Additional file 2.** Research data of this study.

## Data Availability

All data generated or analysed during this study are included in this published article and its supplementary information files. The datasets used and/or analysed during the current study are available from the corresponding author on reasonable request.

## Abbreviations

*Rh. tanguticum*: *Rheum tanguticum* Maxim. ex Balf
PH: Plant height
RL: Root length
RD: Root diameter
LL: Leaf length
LLL: Leaf lobes length
LLP: Leaf lobes percentage
HA: Under canopy habitat
HB: Open habitat
HCA: Hierarchical clustering analysis
PCA: Principle component analysis
OPLS-DA: Orthogonal projections to latent structures discriminant analysis
QC: Quality control
ESI: Electrospray ionization
RT: Retention time
RPT: Response permutation testing
VIP: The variable importance for the projection
ANOVA: Analysis of variance
ADME: Absorption, distribution, metabolism, and excretion
UPLC: Ultra performance liquid chromatography
