# Supplementary material for: Non-target metabolomics revealed the differences between Rh. tanguticum plants growing under canopy and open habitats
Source: BMC Plant Biol. 2021 Feb 27;21:119. doi: 10.1186/s12870-021-02897-8 (PMC7913229; doi:10.1186/s12870-021-02897-8)
Supplement: Supplementary file 1 — Additional file 1: Fig. S1: Total iron current chromatograms of metabolomics analysis in positive scan mode (a) and negative scan mode (b). Fig. S2: Volcano plots (log10 fold change vs. –log10 P -value). Fig. S3: Soil humidity, soil organic carbon contents and soil total nitrogen contents in the under canopy and open habitats. The top and bottom of each box represent 25th and 75th percentiles, the center line indicates the median, and the little hollow squares indicates mean value. The extents of the whiskers show the extent of the data. The asterisk represent significant difference between two groups. Table S1 Compounds accumulated in different habitats [file 12870_2021_2897_MOESM1_ESM.pdf]

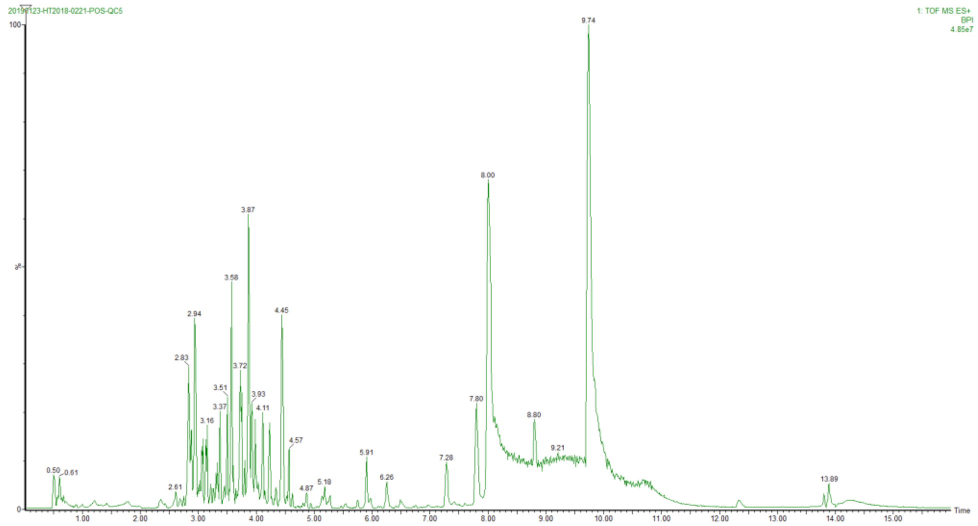

a

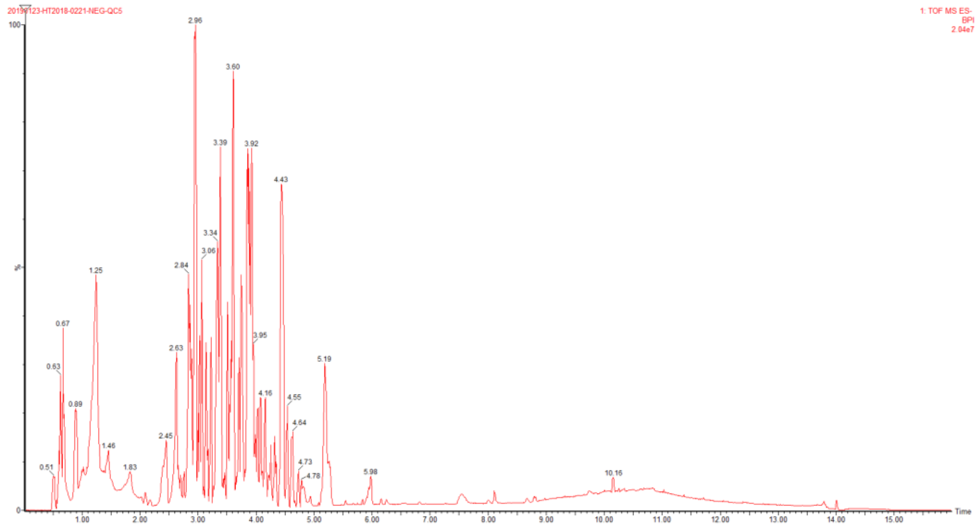

b

Fig. S1: Total iron current chromatograms of metabolomics analysis in positive scan mode (a) and negative scan mode (b).

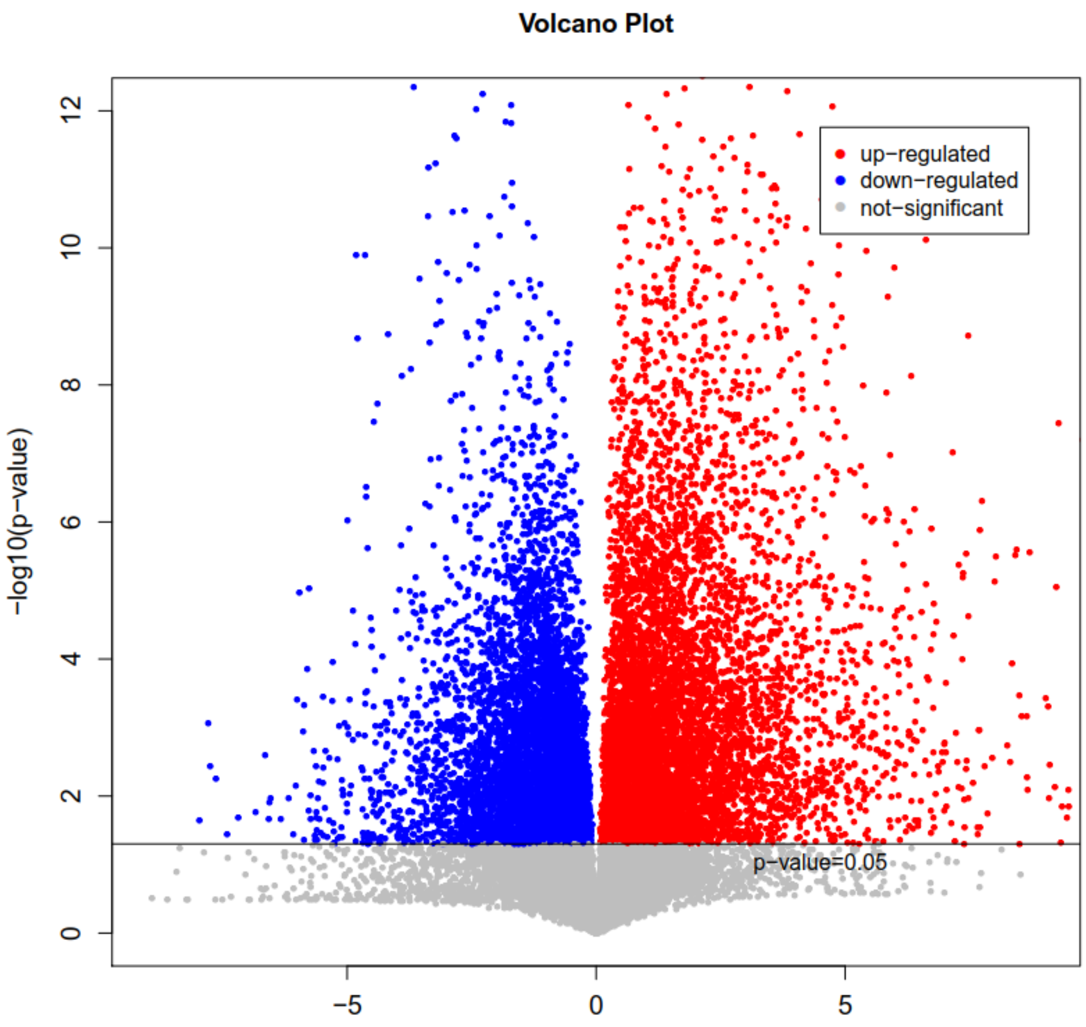

Fig. S2: Volcano plots (log10 fold change vs. –log10 **P** -value)**.**

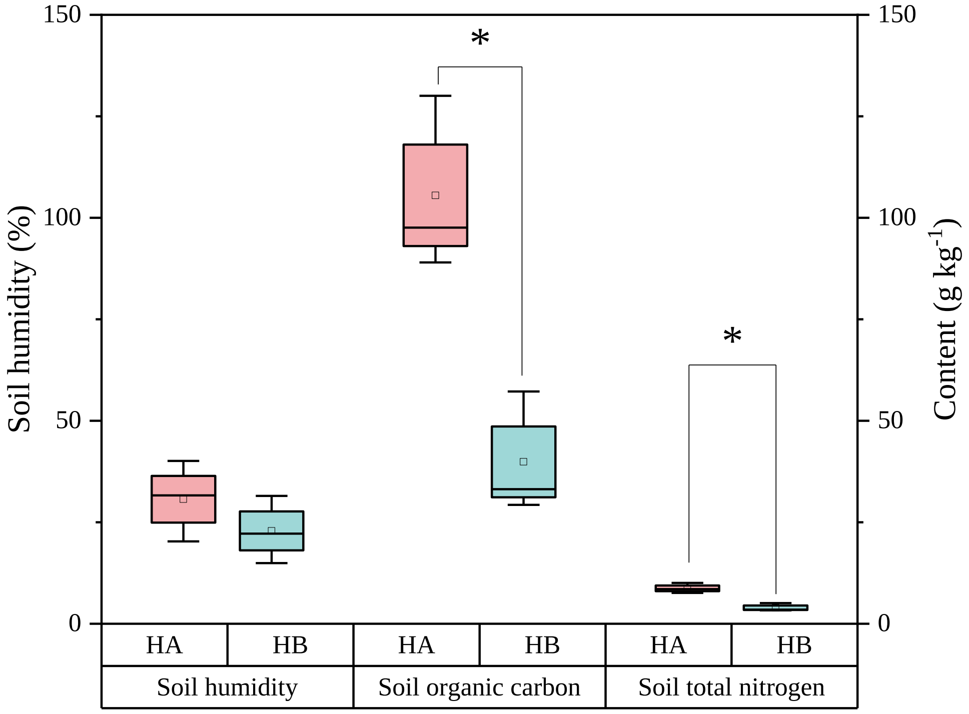

Fig. S3: Soil humidity, soil organic carbon contents and soil total nitrogen contents in the under canopy and open habitats. The top and bottom of each box represent 25th and 75th percentiles, the center line indicates the median, and the little hollow squares indicates mean value. The extents of the whiskers show the extent of the data. The asterisk represent significant difference between two groups.

Table S1 Compounds accumulated more in different habitats

Habitats	Compounds symbol	Compounds name
HA	HA1	(E)-2-Methyl-2-buten-1-ol
HA	HA2	3,4-Dihydro-2H-1-benzopyran-2-one
HA	HA3	Emodinanthranol
HA	HA4	Eugenitol
HB	HB1	1-Hydroxy-3,7-dimethoxyxanthone
HB	HB2	Coumarin

HA: under canopy habitat, and HB: open habitat.
